# Biologic predictors of extension of oligoarticular juvenile idiopathic arthritis as determined from synovial fluid cellular composition and gene expression

**DOI:** 10.1002/art.27284

**Published:** 2010-03

**Authors:** Patricia J Hunter, Kiran Nistala, Nipurna Jina, Ayad Eddaoudi, Wendy Thomson, Mike Hubank, Lucy R Wedderburn

**Affiliations:** 1 University College London Institute of Child HealthLondon, UK; 2 University of ManchesterManchester, UK

## Abstract

**Objective:**

To identify biomarkers in the first synovial fluid (SF) aspirate obtained from children with oligoarticular juvenile idiopathic arthritis (JIA), which could be used to identify children whose disease is likely to extend to a more severe phenotype.

**Methods:**

Patients with recent-onset oligoarticular JIA were identified and grouped according to those whose mild disease persisted (persistent disease) or those whose disease would extend from a mild to more severe phenotype (extended-to-be disease) at 1 year after diagnosis. Flow cytometry was used to delineate differences in the mononuclear cell populations between the first blood sample and first SF aspirate from the same patient and between outcome (persistent versus extended-to-be) groups. Proportions of lymphocytes in the joint were modeled on chemotaxis of lymphocytes to CCL5, using Transwell migration assays. Levels of CCL5 in the SF were quantified by enzyme-linked immunosorbent assay. RNA profiles of SF mononuclear cells were compared between groups using the Affymetrix GeneChip hybridization protocol and hierarchical clustering analyses.

**Results:**

Compared with peripheral blood mononuclear cells, SF mononuclear cells displayed an expansion of CD8+ T cells, reduced proportion of B cells, and expansion of CD16− natural killer cells. The lower CD4:CD8 ratio in the SF was recapitulated in vitro by the observed migration of blood T cells in response to CCL5. Synovial CCL5 levels were higher in children whose disease extended to a more severe phenotype. The CD4:CD8 ratio in the SF was significantly lower in patients with extended-to-be oligoarticular JIA (0.57 compared with 0.90 in the persistent disease group, difference 0.33, 95% confidence interval 0.04–0.62; *P* = 0.009). Gene expression profiling revealed that 344 genes were >1.5-fold differentially expressed between outcome groups (*P* < 0.05), and these included genes associated with inflammation and macrophage differentiation, which showed increased levels in patients with extended disease at 1 year, and genes associated with immune regulation, which showed increased levels in patients with persistent disease at 1 year.

**Conclusion:**

Analyses of the proportions of synovial lymphocytes, levels of CCL5, and differential gene expression yielded potential biomarkers with which to predict the likelihood of extension of oligoarticular JIA to a more severe disease phenotype.

Inflammatory arthritis in children presents with a diverse range of phenotypes, of which the most common is arthritis that starts in ≤4 joints, known as oligoarticular juvenile idiopathic arthritis (JIA) (1). Although sometimes thought of as a benign condition, oligoarticular JIA may, in fact, lead to a wide spectrum of outcomes, ranging from complete remission after discontinuation of medication to development of a severe, extended form of JIA that spreads to involve many joints (≥5 joints). Extended oligoarticular JIA can be highly erosive and destructive and may be difficult to control with conventional disease-modifying antirheumatic drugs (DMARDs), thus requiring long-term treatment with biologic therapies. When oligoarticular JIA remains limited to ≤4 joints, so-called persistent oligoarticular JIA, it is typically relatively easy to control with local intraarticular steroids and nonsteroidal medication (2,3).

A large study of remission rates in different subtypes of JIA found that after ≥4 years of followup, only 31% of children with extended oligoarticular JIA achieved remission after discontinuing their medication, compared with 68% of those with persistent oligoarticular JIA (4). In a long-term retrospective outcome study in adults who had previously been diagnosed as having JIA, those with extended oligoarticular JIA had significantly worse Health Assessment Questionnaire physical function scores than those whose disease had remained persistent (in ≤4 joints) (5). Extension of the disease to ≥5 joints in this form of childhood arthritis has been reported to occur in as high as 50% of patients at 5 years and carries a far higher risk of chronic disability (6). Several clinical factors have been proposed as predictors of extension, such as a high erythrocyte sedimentation rate (ESR), upper-limb involvement, and involvement of >1 joint or symmetric joint involvement at presentation (6,7). Currently, no simple clinical algorithm can be applied to predict extension, but meeting this need would represent a major step forward in the care of children with arthritis.

We have previously shown that once extension of the disease to many joints has occurred, these 2 subtypes of oligoarticular JIA have significantly different immunologic characteristics in the synovial infiltrate. Thus, we demonstrated that the proinflammatory T cell subset that produces interleukin-17 (IL-17), IL-21, and IL-22 (the Th17 subset) is enriched in the joints, compared with the blood, of children with JIA, and that this cell type is found in significantly higher numbers in the joints of children with extended oligoarticular JIA than in those with persistent oligoarticular JIA (8). In contrast, we and others have shown that immunoregulatory T cells (known as Treg cells), which express high levels of CD25 (IL-2 receptor α) and the transcription factor FoxP3, are present at significantly higher numbers in the joints of children with persistent oligoarticular JIA than in those with extended disease (9,10). Interestingly, we also found that these 2 specific T cell subpopulations show a directly reciprocal relationship within the joint (8).

In addition to these cellular correlates, there are significant genetic differences between these 2 subtypes of JIA. Within the HLA region, which codes for multiple genes critical to immune function, the haplotype HLA–DRB1*0801/DQA1*0401/DQB1*0402 is associated with both persistent and extended oligoarticular JIA, although the effect size is greater within the extended disease subgroup, while the HLA–DRB1*1301/DQA1*01/DQB1*06 haplotype confers an increased risk of persistent, but not extended, oligoarticular JIA (11). Similarly, an IL-10 promoter polymorphism that is associated with low IL-10 production (ATA haplotype) has been found to be significantly associated with extended oligoarticular JIA (12).

Given these cellular and genetic associations that are relatively specific for these 2 subtypes of JIA, we reasoned that there may be differences in molecular or cellular features within the inflamed joint that occur early in oligoarticular JIA and that could be predictive of extension from mild to a more severe disease. Once these differences are identified, they might form the basis of a diagnostic test, which, combined with clinical features, might be used to generate an algorithm allowing selection of those children who would need more frequent followup and might require early intervention with DMARDs. Previous studies using gene expression profiling in JIA have focused upon polyarticular disease (13) or have not distinguished between persistent and extended oligoarticular JIA (14). To our knowledge, no previous study has specifically chosen to identify predictors of disease extension, before it occurs, with the use of transcriptome analyses. Such an approach to the development of predictive biomarkers has been highly successful in other fields, notably, oncology (15,16).

In the present study, we therefore tested the hypothesis that synovial fluid (SF) cellular composition, gene expression, and/or cytokine and chemokine profiles could reveal significant differences that might be predictive of the extension of oligoarticular JIA before it occurs. We used SF samples obtained at the first therapeutic aspiration and before use of DMARDs, and we chose early disease extension (within 1 year after diagnosis) as the end point. Of note, we have opted to use the term extended-to-be for those children who were studied at a time when the disease was still limited to ≤4 joints but whose oligoarthritis had extended to a more severe phenotype by 1 year of followup.

## PATIENTS AND METHODS

### Patients and samples

Samples from a total of 38 children (26 female and 12 male) who met the International League of Associations for Rheumatology criteria for oligoarticular JIA (1) and 6 healthy children as controls were included in this study. Patients attended either Great Ormond Street Hospital, London, or the Royal Victoria Infirmary, Newcastle, UK, the latter as part of the Childhood Arthritis Prospective Study (CAPS) (17). The study received approval from the local ethics review committee and the multicenter ethics review committee. Full informed consent was obtained from the parents of each child.

The 38 patients with oligoarticular JIA in this study had a mean age of 8.8 years (range 1.3–13 years) and median disease duration of 7 months (range 2–16 months). All 38 children were undergoing their first knee aspiration of SF, which was clinically indicated in order to receive an intraarticular injection of triamcinolone hexacetonide, and none of the children had yet received methotrexate, steroids, or any other DMARD. SF samples were obtained at the time of clinically indicated arthrocentesis and, where available, samples of peripheral blood (PB) were collected at the same time. Samples were processed within 1 hour of removal from the patient.

PB mononuclear cells (PBMCs) were isolated with density centrifugation using Lymphoprep (Axis-Shield, Oslo, Norway). For preparation of SF mononuclear cells (SFMCs), samples were first treated with 10 units/ml hyaluronidase (Sigma, Poole, UK) for 30 minutes at 37°C, before isolation with Lymphoprep density centrifugation. For some patients, samples of plasma or SF were also obtained for the analysis of cytokines and chemokines. These samples were spun to remove cells, and were frozen at −80°C within 1 hour after removal from patients.

### Analysis by flow cytometry

Standard 5-color flow cytometry was performed for surface markers using directly labeled monoclonal antibodies against the following human proteins: CD3–phycoerythrin (PE)-Cy7 (UCHT1; Southern Biotechnology, Birmingham, AL), CD3–allophycocyanin (APC) (S4.1; Caltag, Burlingame, CA), CD4–peridinin chlorophyll A protein (PerCP) (L200; BD Biosciences, San Diego, CA), CD4-APC (S3.5; Southern Biotechnology), CD8–fluorescein isothiocyanate (FITC) (G42-8; BD Biosciences), CD8-PE (DK25; Dako, Glostrup, Denmark), CD8–PE-Cy7 (RPA-T8; BD Biosciences), CD8-APC (RTF8; Southern Biotechnology), CD13-APC (WM15; BioLegend, San Diego, CA), CD14-PerCP (ΜΦP9; BD Biosciences), CD16–PerCP-Cy5.5 (3G8; BD Biosciences), CD19-FITC (HIB19; BD Biosciences), CD25-PE (ACT-1; Dako), CD25–PE-Cy5 (M-A251; BD Biosciences), CD56-PE (B159; BD Biosciences), CD195-PE (2D7/CCR5; BD Biosciences), and T cell receptor γ/δ–FITC (11F2; BD Biosciences).

The following intracellular proteins were detected by flow cytometry, following fixation and permeabilization of the cells: FoxP3-APC (PCH101; eBioscience, San Diego, CA), Ki-67–FITC (B56; BD Biosciences), and IL-17–Alexa Fluor 647 (64CAP17; eBioscience). For detection of FoxP3, buffers from the FoxP3 Staining Set (eBioscience) were used. For detection of Ki-67 and IL-17, cells were fixed with 4% paraformaldehyde (BDH Chemicals, Poole, UK) in phosphate buffered saline (PBS) (Sigma) and permeabilized with 0.1% saponin in PBS containing 1% fetal calf serum (Invitrogen, Renfrew, UK) and 0.1% sodium azide (Sigma). For analysis of IL-17 production by T cells, the SFMCs or PBMCs were cultured for 3 hours in the presence of 50 ng/ml of phorbol myristate acetate (Sigma), 500 ng/ml of ionomycin (Sigma), and 5 μg/ml of brefeldin A (Sigma), before analysis by intracellular staining and flow cytometry as described above.

Levels of apoptosis in cell populations were estimated by detecting active caspases using the Vybrant FAM Poly Caspase Assay Kit (Molecular Probes, Eugene, OR). VAD (Val-Ala-Asp) binds to the groove in most caspases (including caspase 1 and caspases 3 through 9), and these are exposed by cleavage to their active forms (18). The FLICA reagent combines VAD with FMK to create a covalent link with the target caspase, and FAM is used for fluorescence detection. Cells were resuspended in RPMI 1640 (Invitrogen) at 1 × 10^6^/ml, incubated at 37°C in 5% CO_2_ for 1 hour with FLICA reagent, washed with the kit buffer, and stained for CD3, CD4, and CD8. PBMCs that had been stimulated with anti-CD3/anti-CD28 beads (Miltenyi Biotech, Bergisch Gladbach, Germany) for 4 days followed by 20-hour treatment with brefeldin A (Sigma) were used as a positive control for caspase activity.

Flow cytometric data were collected on an LSRII (BD Biosciences) or Cyan ADP (Beckman Coulter, Fullerton, CA); from 100,000 to 200,000 events were collected for each condition, and cells were gated according to scatter properties. Flow cytometric data were analyzed using FlowJo software (TreeStar, Ashland, OR).

### Enzyme-linked immunosorbent assay (ELISA)

Detection of CCL5 in the plasma and SF was performed using an ELISA (R&D Systems, Abingdon, UK) according to the manufacturer's instructions.

### Chemotaxis assay

PBMCs were allowed to adhere to plastic for 3 hours at 37°C in 5% CO_2_ in RPMI/5% human AB serum in order to deplete monocytes and enrich for lymphocytes. Nonadherent cells at a starting concentration of 5 × 10^6^/ml were exposed to 20–500 ng/ml CCL5 (R&D Systems) in RPMI/0.5% bovine serum albumin (BSA) or RPMI/0.5% BSA alone in the lower chamber of a 5-μm–pore, polycarbonate-filter Transwell chamber (Corning Life Sciences, Schiphol-Rijk, The Netherlands). After 90 minutes, migrated cells were recovered and stained with anti-human CD3, CD8, and CD4 antibodies. Immediately prior to analysis of the data by flow cytometry, 20,000 fluorescence-activated cell sorter counting beads (Perfect Count; Cytognos, Salamanca, Spain) were added per sample. The numbers of cells detected per sample were standardized relative to the bead numbers.

### Gene expression profiling

SFMCs were thawed and total RNA was prepared from 2–5 × 10^6^ cells using RNeasy spin columns (Qiagen, Hilden, Germany). The quality and purity of the RNA was assessed using a Bioanalyzer 2100 (Agilent Technologies, Palo Alto, CA). Complementary DNA and subsequent complementary RNA (cRNA) synthesis was then performed as previously described (19). Purified cRNA transcripts were fragmented and hybridized to human U133 plus 2.0 GeneChips according to Affymetrix standard protocols (see http://www.affymetrix.com). Signal values were calculated using MAS 5.0, with results scaled to 100 and normalized to the median value, prior to analysis with GeneSpring GX 10 software (Agilent Technologies). Probe sets were excluded if the signal strength did not significantly exceed background values and if expression did not reach a threshold value for reliable detection (based on the relaxed Affymetrix MAS 5.0 probability of detection; *P* < 0.1 [20]) in 5 of 21 samples.

A list of probe sets in which the difference in mean expression levels between the 2 patient groups was more than 1.5-fold was statistically analyzed by the Student's *t*-test with Welch's correction, and a *P* value cutoff of 0.05 was applied for the level of significance. Functional categories for corresponding genes were assigned using gene ontology terms from the Gene Ontology Consortium (see www.geneontology.com). Hierarchical clustering was performed using average linkage. Complete gene expression data for each patient are available on the GEO database (GEO accession no. GSE15083; www.ncbi.nlm.nih.gov).

### Statistical analysis

All analyses of the data, including logistic regression models, were performed in SPSS version 15 (SPSS, Chicago, IL). *P* values less than 0.05 were considered significant.

## RESULTS

### Clinical characteristics

In this study of the first joint aspirate obtained from children with oligoarticular JIA, we defined the composition of the mononuclear cell populations in the SF compared with the PB. We then looked for demonstrable differences in SFMC composition in children whose arthritis would remain mild (persistent oligoarticular JIA) compared with those whose disease would extend to a more severe phenotype (extended-to-be oligoarticular JIA) at 1 year after diagnosis. At the time of sampling, there were no significant differences in the clinical characteristics between these 2 outcome groups (Table 1).

**Table 1:** Clinical characteristics of the patients with known outcome at 1 year*

Outcome	Persistent oligoarticular JIA (n = 21)	Extended-to-be oligoarticular JIA (n = 11)
Female, no. (%)	14 (67)	8 (73)
Age at sampling, mean ± SD years	6.8 ± 3.7	5.1 ± 3.5
Disease duration, mean ± SD months	7.4 ± 3.7	8.1 ± 3.9
Erythrocyte sedimentation rate, mean ± SD mm/hour	21 ± 17	29 ± 23
Receiving MTX or prednisolone prior to or at the time of sampling, no.	0	0

* JIA = juvenile idiopathic arthritis; MTX = methotrexate.

### Differences in composition of mononuclear cells in the synovial exudate compared with the peripheral blood in children with oligoarticular JIA

**Figure 1:**
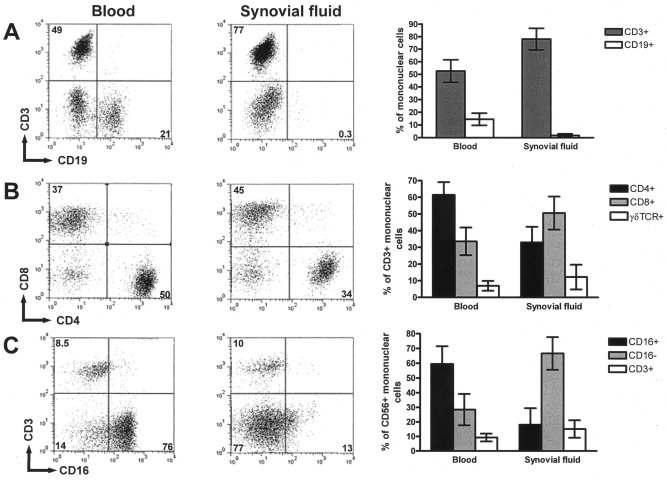
Differences in mononuclear cell subsets in the synovial fluid compared with peripheral blood of children with oligoarticular juvenile idiopathic arthritis. Flow cytometry was used to enumerate proportions of **A**, T cells versus B cells from the live cell gate, **B**, CD4+ and CD8+ T cell subsets from the CD3+ live cell gate, as well as T cell receptor γ/δ–bearing (γδTCR) cells detected with a specific antibody, and **C**, natural killer cell subsets from the CD56+ live cell gate. Representative results are shown, in flow cytometry plots (left) and quantitatively as the mean ± SD proportion of each cell subset in 14 paired patient samples (right).

Flow cytometry was used to compare the proportions of mononuclear cell subsets between the PB and aspirated SF from children with oligoarticular JIA. The most prominent differences were observed in the paucity of B cells in the SF (mean ± SD 1.8 ± 1.3% versus 14.5 ± 4.8% in the blood) (Figure 1A) and the predominance of CD8 T cells in the SF (Figure 1B), so that there was a reversal of the typical CD4:CD8 T cell ratio observed in the blood of these children (21). Within the natural killer (NK) cell subset, identified as expressing CD56, we observed a preponderance of CD16− cells in the SF (Figure 1C). The majority of blood-borne NK cells express the Fc receptor. The down-regulation of CD16 on NK cells in an inflamed site has been previously described in patients with rheumatoid arthritis (RA) (22).

### Correlation of reversal of the CD4:CD8 ratio in synovial T cells early in disease with likelihood of progression to a more severe disease phenotype

**Figure 2:**
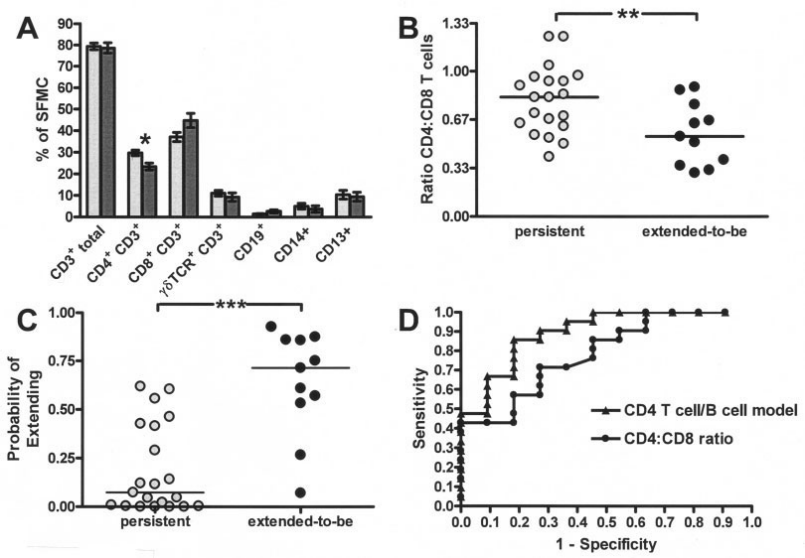
Differences in the proportions of synovial fluid mononuclear cell (SFMC) subsets in patients with persistent oligoarticular juvenile idiopathic arthritis (JIA) (n = 21) compared with those with extended-to-be oligoarticular JIA (n = 11), and the utility of these measures as predictors of outcome. **A**, Proportions of T cell, B cell, monocyte/macrophage (CD14+), and total myeloid cell (CD13+) subsets, derived from flow cytometry, in patients with persistent oligoarticular JIA (light gray bars) compared with patients with extended-to-be disease (dark gray bars). Results are the mean ± SEM percentage relative to that of total live cells. **B**, CD4:CD8 T cell ratios, derived from flow cytometry, in patients with persistent versus extended-to-be oligoarticular JIA. Circles indicate individual samples; horizontal lines indicate the median. **C**, Probability of disease extension in a logistic regression model using the proportions of CD4+ and CD19+ lymphocytes to predict the outcome, which was correctly predicted in 27 (84%) of 32 patients. Circles indicate individual patients; horizontal lines indicate the median. **D**, Receiver operating characteristic curves comparing the CD4:CD8 ratio (area under the curve 0.79) with the CD4+ T cell–B cell model derived from logistic regression (area under the curve 0.90). ∗ = *P* < 0.05; ∗∗ = *P* < 0.01; ∗∗∗ = *P* < 0.001, between outcome groups, by Mann-Whitney U test.

We compared the proportions of SF T cell, B cell, NK cell, monocyte/macrophage, and dendritic cell subsets between the patient group whose disease had remained persistent (mild disease in <5 joints) (n = 21) and those whose disease had extended to ≥5 joints (n = 11) at 1 year after diagnosis. A statistically significant difference was observed in the proportion of the total SFMCs that were CD4+ T cells (Figure 2A). While both groups had the same proportion of SF T cells overall (Figure 2A), the CD4:CD8 ratio was lower in the group with extended disease at 1 year (difference 0.33, 95% confidence interval [95% CI] 0.038–0.62; *P* = 0.009) (Figure 2B).

Since the CD4:CD8 ratio is a simple laboratory measure that can be routinely applied, we considered its merit as a predictive test for the likelihood of extension of oligoarticular JIA. Using a receiver operating characteristic curve, a ratio of 0.67 was established as the optimal cutoff value for maximizing the sensitivity and specificity of the test (Figure 2D). Thus, in our data set, a patient with a CD4:CD8 ratio of lower than 0.67 was 2.5 times more likely (95% CI 1.16–5.4) to experience disease extension to a more severe phenotype.

Joint cell proportions further increased the predictive power in a logistic regression model that used B cell percentages in addition to CD4 T cell subset percentages. The proportion of B cells was higher, but not significantly higher, in the group with extended disease compared with the group with persistent disease at 1 year (Figure 2A). However, the regression model that combined B cell and CD4+ T cell proportions correctly assigned 27 (84%) of 32 patients (Figure 2C). Factoring in the proportions of other cell subsets, including the CD8+ T cell proportions, improved the accuracy of prediction, but these did not contribute significantly (at a level of *P* < 0.05) to the model. In a separate regression analysis, the clinical parameters of age, disease duration, ESR, and joint count at the time of sampling neither improved the model nor were predictive of the outcome. (The equation used to calculate the probability of disease extension using the CD4 T cell/B cell model is available from the corresponding author upon request).

We have previously shown that once disease extension occurs, there are significant differences in the proportions of both Treg cells and Th17 cells in the joint (8,9). We therefore examined the Treg cell (FoxP3+CD4+) and IL-17+CD4+ T cell subsets in the 2 disease outcome groups. Neither the proportion of Treg cells nor the proportion of Th17 cells, measured at presentation, was predictive of disease extension, although the reciprocal relationship between the numbers of these 2 cell types was maintained (see Supplementary Figure 1, available on the *Arthritis & Rheumatism* Web site at http://www3.interscience.wiley.com/journal/76509746/home).

### Matched rates of cell turnover between synovial CD4 and CD8 T cells

**Figure 3:**
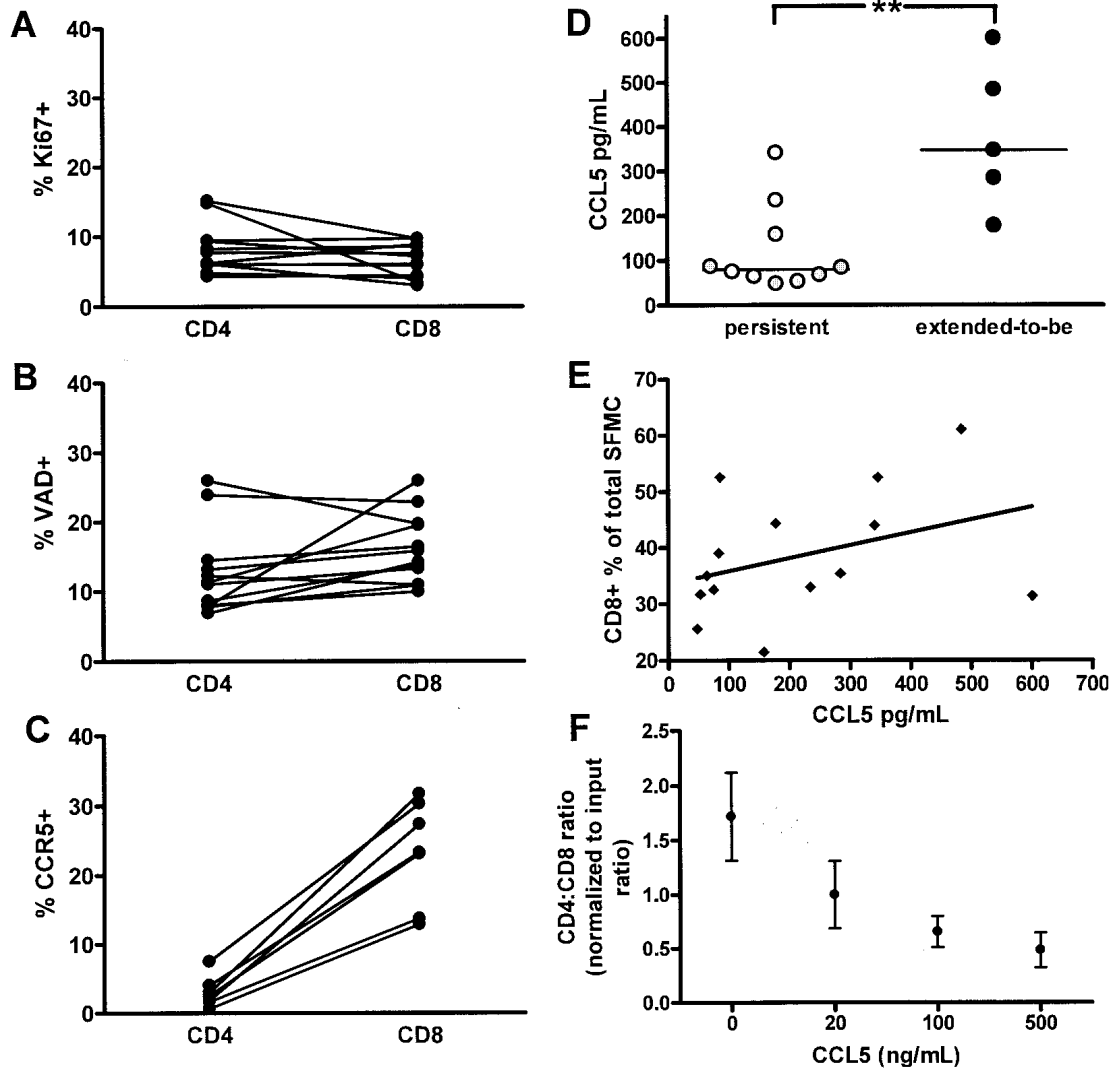
Measures of proliferation, apoptosis, and migration of T cell subsets in the synovial fluid (SF) of patients with oligoarticular juvenile idiopathic arthritis. **A–C**, SF (n = 12) (**A** and **B**) and peripheral blood (n = 7) (**C**) CD4+ and CD8+ T cells were compared for **A**, intracellular expression of Ki-67, **B**, binding of VAD tripeptide to active caspases, and **C**, surface expression of CCR5 as determined by flow cytometry gated on live, CD3+ cells. **D**, The levels of CCL5 (RANTES) in the SF (n = 10 with persistent disease, n = 5 with extended-to-be disease) were measured by enzyme-linked immunosorbent assay. Circles indicate individual patients; horizontal lines indicate the median. **E**, SF levels of CCL5 were compared with CD8+ T cell levels as a percentage of the total live SF mononuclear cells (SFMC) from 14 patients (r^2^ = 0.13, *P* = 0.21). **F**, Migration to CCL5 lowers the CD4:CD8 ratio, as determined in a Transwell assay using nonadherent peripheral blood mononuclear cells from healthy adults. Bars show the mean ± SD results from 3 individual samples. ∗∗ = *P* < 0.01 by Mann-Whitney U test.

Since we were able to establish the predominance of CD8 T cells in the joint and its implications in predicting the disease course in children with oligoarticular JIA, we wanted to investigate the cellular dynamics that are potentially driving CD8 T cell accumulation. We hypothesized that CD8 T cells might be expanding through proliferation or undergoing apoptosis to a lesser extent compared with CD4 T cells. Ki-67 is an antigen that is expressed during the S and M phases of the cell cycle, and thus can be an indicator of the fraction of the T cell population undergoing cell division (23). No difference in the proportion of Ki-67+ cells between CD4 T cells and CD8 T cells from the same SFMC samples was observed (Figure 3A). Similarly, we estimated apoptotic activity by detecting activated caspases using the VAD-FAM reagent. We observed no significant difference in VAD+ cells between CD4 SF T cells and CD8 SF T cells (Figure 3B). Therefore, the observed dominance of CD8 T cells over CD4 T cells in the joint could not be explained by differences in cell death or proliferation.

### Contribution of synovial chemokine migration to the altered CD4:CD8 ratio within the joint

We considered mechanisms that might contribute to enrichment for CD8 T cells through chemoattraction from the blood to the inflamed joint. As shown in Figure 3C, CCR5/CD195 was found to be expressed on a higher proportion of CD8 T cells compared with CD4 T cells, similar to observations in the blood of healthy adults and children (24) as well as the results of our previous study (25). We and others have described a role for CCL5 (RANTES), a ligand of CCR5, in JIA, and indeed, high levels in the blood and SF have been reported to be a predictor of disease flares (26,27). As shown in Figure 3D, the levels of CCL5 were found to be significantly higher in the SF of patients in the extended-to-be disease group compared with patients whose disease remained persistent. The SF CCL5 levels showed a weak correlation with SF CD8+ T cell proportions (Figure 3E), suggesting that CCL5 could play a role in the enrichment for CD8 T cells in the joint.

To test the effect of CCL5 on T cell migration in vitro, nonadherent PBMCs from healthy adults were exposed to increasing concentrations of CCL5 in a Transwell assay. CCL5 was able to enrich for CD8+ T cells to a greater extent than that for CD4+ T cells, thus decreasing the CD4:CD8 ratio in a dose-dependent manner (Figure 3F).

### Differential SFMC gene expression in children with extended-to-be disease

**Figure 4:**
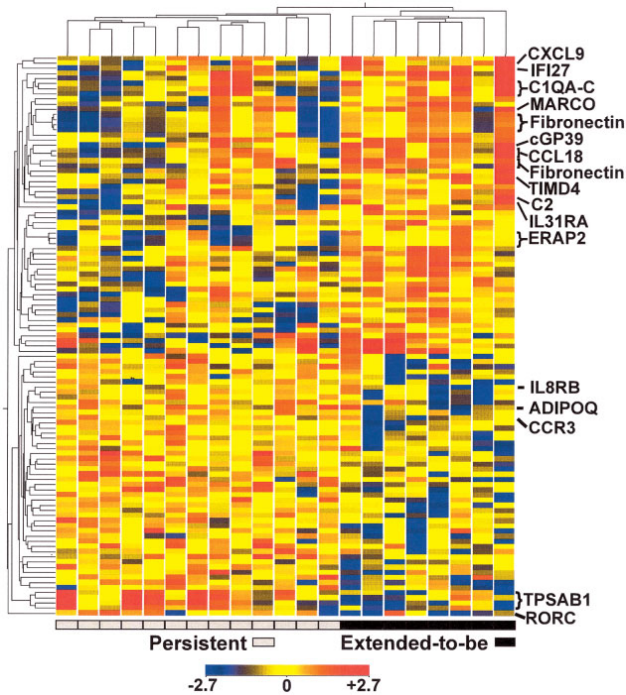
Hierarchical clustering analysis of genes differentially expressed in synovial fluid mononuclear cells of patients with persistent oligoarticular juvenile idiopathic arthritis (JIA) (n = 13) compared with patients with extended-to-be oligoarticular JIA (n = 8). The heat map shows genes (as labeled to the right of the map) that were 2-fold differentially expressed at a significance level of *P* < 0.05, as determined by Student's *t*-test with Welch's correction. Each row represents a different probe set, and each column represents an individual patient sample. The normalized expression level for each gene and each patient is indicated by colors, with red, yellow, and blue reflecting expression levels greater than, equal to, or less than the mean value, respectively, for all of the patients. The boxes below the cluster indicate patients whose disease persisted versus those whose disease had extended at 1 year after diagnosis.

Microarray-generated gene expression levels were obtained in 13 SFMC samples from children with persistent disease and 8 SFMC samples from those with extended-to-be disease. A total of 362 probe sets covering 344 individual genes were at least 1.5-fold differentially expressed at a significance level of *P* < 0.05 (for the annotated probe sets, see Supplementary Table 1, available on the *Arthritis & Rheumatism* Web site at http://www3.interscience.wiley.com/journal/76509746/home). The expression of 155 genes was increased and the expression of 189 genes was decreased in patients with extended disease compared with patients with persistent disease at 1 year. Probe sets with more than 2-fold gene expression were clustered, by average linkage analysis, along with each outcome group (Figure 4).

Some of the differentially expressed genes associated with immune function and inflammation are listed in Table 2 (also labeled in Figure 4). In general, genes encoding proteins with involvement in inflammation tended to be more highly expressed in the extended-to-be disease group, and genes associated with immune regulation were more highly expressed in the persistent disease group. One interesting exception was the increased expression of RORC (retinoic acid–related orphan receptor C), a transcription factor involved in the differentiation of proinflammatory IL-17–secreting cells (28), in the persistent disease group.

**Table 2:** Genes differentially expressed in synovial fluid mononuclear cells of patients with extended-to-be oligoarticular juvenile idiopathic arthritis compared with those with persistent disease at 1 year after diagnosis

Functional category, gene name	Symbol	Fold change*
Cell surface protein		
Interferon-inducible 27	IFI27	4.2
Macrophage receptor with collagenous structure	MARCO	3.1
Interleukin-31 receptor α	IL31RA	2.7
T cell immunoglobulin and mucin domain–containing 4	TIMD4	2.1
Interferon-α–inducible protein	IFI6	1.8
Solute carrier family 31, member 2	SCL31A2	1.7
CD8β	CD8B1	1.6
CXCR6	CXCR6	−1.6
Vasoactive intestinal peptide receptor 1	VIPR1	−1.7
Leukocyte-associated Ig-like receptor 2	LAIR2	−2.0
CXCR2 (interleukin-8 receptor β)	IL8RB	−2.3
CCR3	CCR3	−2.7
Cytokine or chemokine		
CCL18	CCL18	3.0
CXCL9	CXCL9	3.0
Interferon-γ	IFNG	1.7
Small inducible cytokine subfamily E1	SCYE1	1.5
Complement		
C1q, C chain	C1QC	4.0
C1q, B chain	C1QB	3.5
C1q, A chain	C1QA	3.1
C2	C2	3.2
Nuclear receptor or protein		
Nuclear receptor subfamily 4, group A2	NR4A2	2.3
Activating transcription factor 3	ATF3	1.9
Runt-related transcription factor 1	RUNX1	1.7
Retinoic acid–related orphan receptor C	RORC	−3.4
Cytoplasmic protein		
Fatty acid binding protein 3	FABP3	2.3
Endoplasmic reticulum aminopeptidase 2	ERAP2	2.2
Sma and Mad (*Drosophila*)–related protein homolog 3	SMAD3	−1.7
Extracellular mediator		
Chitinase 3–like 1 (cartilage glycoprotein 39)	CHI3L1	4.2
Fibronectin 1	FN1	2.8
Adiponectin	ADIPOQ	−2.5
α-tryptase or β-tryptase 1	TPSAB1	−6.0

* Fold change is the ratio of the mean gene expression value in the extended-to-be disease group to that in the persistent disease group. Positive values denote higher expression in the extended-to-be disease group, and negative values indicate higher expression in the persistent disease group.

A subset of genes that were more highly expressed in the extended-to-be disease group included markers of macrophage differentiation to both M1 effector cell types (CXCL9, SLC31A2, ATF3) and M2 effector types (CCL18, fibronectin) (29). Several transcripts of genes for components of the complement pathway, also likely derived from macrophages, were overrepresented in the extended-to-be disease group. The proportion of monocyte/macrophages, as identified by coexpression of CD13 and CD14 in the SFMC samples, did not significantly differ between the persistent and extended-to-be disease sample sets that were subjected to microarray analysis or in the complete patient groups (Figure 2A). Cartilage glycoprotein 39 (chitinase 3–like 1), which is found in the serum and synovium of patients with RA and patients with osteoarthritis, shows a tendency to be correlated with disease severity (30), and therefore, it was interesting to find more of its transcript in the patients whose disease extended to oligoarticular JIA in this study.

There was good agreement between gene expression and protein measurements for the CD8 antigen. This antigen was more highly expressed in the extended-to-be disease group, in whom there was also a higher proportion of CD8+ T cells. Similarly, higher levels of interferon-γ (IFNγ) messenger RNA corresponded to a trend toward proportionally more IFNγ in the SF of the patients with extended-to-be disease, as detected by Luminex assay (results not shown).

## DISCUSSION

This study was performed to identify predictors of the future worsening of disease in children with an initially benign arthritis, by screening for molecules in the affected joints. Our results revealed biomarker candidates with previously described roles in inflammatory or regulatory processes, as well as novel factors whose role in JIA are not yet clearly defined.

This report documents the dominance of CD8 T cells in the joint and the corresponding reversal of the CD4:CD8 ratio in the blood, which is typically a ratio of between 1.5 and 3 in healthy individuals (31) but was <1 in the majority of analyzed SF samples from children with oligoarticular JIA. In this study, the extent to which this reversal occurs in the joint is a predictor of unfavorable outcome. Determination of the CD4:CD8 ratio is a simple procedure and could be easily adopted as a prognostic test, if validated in a larger study group. Logistic regression analyses of all mononuclear cell types revealed that B cells, even in their reduced proportions in the SF compared with that in the blood, can also function prognostically and in a manner independent of the CD4:CD8 ratio.

The predominance of CD8 T cells in the synovial tissue of patients with JIA, and in the oligoarticular subtype in particular, has been previously described (32). In adult patients with RA, CD4+ T cells are retained in the synovium, while CD8+ T cells accumulate in the SF (33). Unlike the findings in patients with type I diabetes, in whom CD8 T cells participate in the destruction of pancreatic islet cells through specific lysis (34), no clear role for the cytotoxic lymphocyte has yet been demonstrated in the joint. Furthermore, synovial T cells have been reported to be anergic ex vivo (35). Since the level of turnover of synovial T cells did not differ between subsets, our results support a model in which the distribution of CD4 and CD8 T cells in the joint is a reflection of the cytokine and chemokine milieu attracting migration into the joint. Our results also support a role for CCL5 (RANTES), which was demonstrated to influence migration of CD8 T cells over CD4 T cells and to be a predictor of the extension of oligoarticular JIA. Therefore, although we cannot exclude the possibility of a distinct role for CD8 T cells, a low CD4:CD8 ratio may simply be an indirect measure of the effects of chemokines on migration to the joint. Once in the joint, CD8 T cells, and not CD4 T cells, produce CCL5 (26). This may therefore provide a positive feedback loop enhancing the selective migration of CD8 T cells.

Further support for a role of CCL5 derives from the finding of a higher expression of TPSAB1, the gene encoding β-tryptase, in patients with persistent disease. Beta-tryptase is a serine protease whose proarthritic potential (demonstrated in mice) (36) is thought to be due to its similarity to other matrix-destroying proteinases (37). However, Pang et al recently described a role for β-tryptase in the abrogation of CCL5 by specific cleavage (38). Thus, this protein could play a role in limiting chemokine activity by cleavage within the joint.

The profile of differentially expressed genes between patients whose mild disease persisted and those whose disease became worse gives a snapshot of early disease in which the shift toward proinflammatory versus antiinflammatory mechanisms has already been made. A significant subset of the genes expressed in the extended-to-be disease group overlapped with genes expressed in activated macrophages in vitro (29). Differentiation of macrophages, particularly of the M1 type, is associated with greatly increased transcriptional and metabolic activity (29). Since the proportions of monocyte/macrophage-phenotyped cells did not significantly differ in the samples subjected to microarray analysis, it is possible that the level of activation or differentiation in these cells was higher in the patients whose disease would later extend. These macrophage-associated genes, together with complement and cartilage glycoprotein 39, form a group of genes that are predictive of disease extension and link oligoarticular JIA to more severe forms of JIA, such as the polyarticular and systemic-onset forms (39–41).

Genes that were more highly expressed in children with persistent oligoarticular JIA revealed factors with roles in immune regulation that were not previously examined in JIA. Adiponectin and the vasoactive intestinal peptide receptor both have antiinflammatory signaling properties that are correlated with less severe RA and with the amelioration of collagen-induced arthritis in mice (42–46). Our results implicate new pathways to disease control that may act independently or in concert with the previously characterized activity of Treg cells. It was interesting that neither the Treg cell proportions nor the FoxP3 protein levels in these cells were predictive of the outcome in this study. However, the association of SMAD3 with a more favorable outcome is consistent with its role as a transducer of the transforming growth factor β (TGFβ) signal that leads to FoxP3 expression and regulatory function (47). Similarly, proportions of Th17 cells were not predictive in this patient group, and RORC, the transcription factor associated with Th17 cell development, was more highly expressed in the patients whose disease persisted. Similar to TGFβ, the transcription factor RUNX1 has been linked to both FoxP3 and RORC transcriptional activity (48,49). RUNX1 and SMAD3, which were associated with disease extension and disease persistence, respectively, could therefore be functioning to modulate the transcriptome to tip the balance in favor of heightened inflammation or a more quiescent environment in the joint.

A possible limitation of using synovial cells for this analysis is that not all patients will have an SF aspirate available or may not have undergone synovial aspiration. However, previous studies comparing gene expression profiling on peripheral blood cells in JIA have been unable to distinguish between persistent oligoarticular JIA and oligoarticular JIA that has already extended (at that time, referred to as polyarticular juvenile RA) (14). For this reason and based on our previous findings indicating differences in the SF between those with persistent oligoarticular JIA and those with extended disease, we chose to focus on synovial cells herein. Findings in other studies that have attempted to identify clinical predictors of disease extension have suggested that the pattern and number of affected joints at presentation may be useful for prediction. All of the subjects in this study had knee arthritis, and therefore, they may not represent a fully representative cross-section of all types of oligoarticular JIA. In addition, the study size may have been too small to detect such a predictive value of these clinical factors. In our analysis in this group, adding such clinical indices into the model did not improve our ability to predict those whose disease would extend. In addition, we focused on outcome at only 1 year, since this study aimed to uncover predictors of early extension; it is, of course, possible that the children in the persistent oligoarticular JIA group in this study might later experience an extension of arthritis into 5 or more joints. However, we suggest that predictors of early extension may well be useful clinical/prognostic tools in the management of oligoarticular JIA.

Thus, in this study, we have proposed new ways to identify children at risk of developing a more severe form of JIA after an initial diagnosis of mild disease, by examining transcript and protein levels in the SF. These findings now deserve further investigation in prospective studies. Although it is unlikely that any single measurement will individually discriminate between these 2 outcomes, it is possible that a biomarker set of strong predictors could provide a valuable prognostic tool for this type of childhood arthritis.

## AUTHOR CONTRIBUTIONS

All authors were involved in drafting the article or revising it critically for important intellectual content, and all authors approved the final version to be published. Dr. Hunter had full access to all of the data in the study and takes responsibility for the integrity of the data and the accuracy of the data analysis.

**Study conception and design.** Hunter, Eddaoudi, Hubank, Thomson, Wedderburn.

**Acquisition of data.** Hunter, Nistala, Jina.

**Analysis and interpretation of data.** Hunter, Nistala, Eddaoudi, Hubank, Wedderburn.
