# Supplementary material for: Biologic predictors of extension of oligoarticular juvenile idiopathic arthritis as determined from synovial fluid cellular composition and gene expression
Source: Arthritis Rheum. 2010 Mar;62(3):896–907. doi: 10.1002/art.27284 (PMC2860766; doi:10.1002/art.27284)
Supplement: Supplementary file 1 [file art0062-0896-SD1.pdf]

Supplementary data Hunter et al 2010

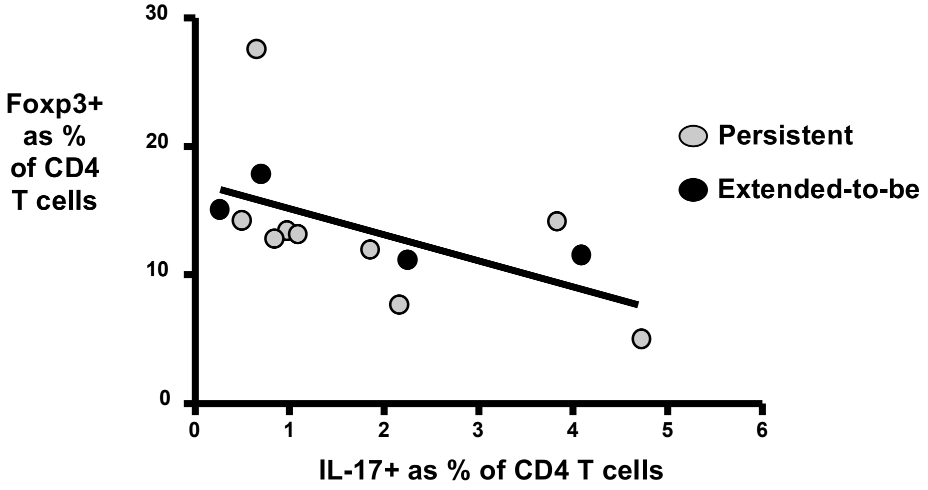

Supplementary Figure. Inverse relationship between Treg and Th17 cell numbers in CD4+ T cells of patients with OA-JIA. Intracellular cytokine detection of IL-17 and nuclear detection of Foxp3 was performed on a subset of patients listed in Table 1. Dot plot shows persistent OA-JIA (n=9) (light grey) and extended-to-be OA-JIA (n=4) (black). r^2= 0.33 P=0.04

Supplementary Table

Probe Set ID	*P*-value	FC[1]	regulation[2]	Unigene ID	Gene Symbol
207156_at	6.98E-04	1.51	down	Hs.51011	HIST1H2A-M
221171_at	7.41E-04	1.63	up	Hs.709801	RP4-692D3.1
224657_at	8.34E-04	1.60	up	Hs.605445	ERRFI1
205019_s_at	9.32E-04	1.66	down	Hs.348500	VIPR1
226113_at	9.86E-04	1.55	down	Hs.293798	ZNF436
205398_s_at	1.02E-03	1.69	down	Hs.618504	SMAD3
203019_x_at	1.31E-03	1.55	down	Hs.22587	SSX2IP
226213_at	1.65E-03	2.09	down	Hs.118681	ERBB3
232504_at	2.01E-03	2.43	down	Hs.604728	LOC285628
228630_at	2.28E-03	1.51	down	Hs.654730	ZNF84
219315_s_at	2.29E-03	2.04	down	Hs.459652	TMEM204
232752_at	2.32E-03	1.70	down	Hs.599785	FLJ31814
212089_at	2.35E-03	1.68	up	Hs.594444	LMNA
241216_at	2.37E-03	1.74	up	Hs.688533	
213430_at	2.52E-03	1.58	up	Hs.7972	RUFY3
227500_at	2.62E-03	1.72	down	Hs.623974	FBXL18
237245_at	3.25E-03	2.05	down	Hs.86761	
215382_x_at	3.25E-03	6.00	down	Hs.405479	TPSAB1
209109_s_at	3.46E-03	2.76	down	Hs.43233	TSPAN6
228081_at	3.55E-03	1.58	down	Hs.655742	FLJ35319
228099_at	3.62E-03	1.61	down	Hs.180257	ZNF550
219049_at	3.76E-03	1.60	down	Hs.655166	CSGALNACT1
227351_at	3.96E-03	1.57	down	Hs.498890	C16orf52
1553417_at	4.12E-03	2.67	down	Hs.376151	C11orf44
35148_at	4.29E-03	2.32	down	Hs.25527	TJP3
203222_s_at	4.37E-03	1.68	down	Hs.197320	TLE1
211021_s_at	4.52E-03	1.71	down	Hs.9347	RGS14
235893_at	4.61E-03	1.77	down	Hs.633613	FLJ34312
201212_at	4.72E-03	1.77	up	Hs.18069	LGMN
238070_at	4.92E-03	1.52	up	Hs.191164	CHD1L
230235_at	4.97E-03	1.60	down	Hs.602444	
222360_at	5.33E-03	1.75	down	Hs.440776	DPH5
209396_s_at	5.40E-03	4.24	up	Hs.382202	CHI3L1
221052_at	5.42E-03	1.58	down	Hs.144439	TDRKH
242139_s_at	5.60E-03	1.68	down		
208228_s_at	5.66E-03	1.76	down	Hs.533683	FGFR2
1554600_s_at	5.87E-03	1.61	up	Hs.594444	LMNA
1552280_at	6.13E-03	2.06	up	Hs.334907	TIMD4
219494_at	6.14E-03	1.67	down	Hs.30561	RAD54B
228708_at	6.25E-03	1.51	down	Hs.25318	RAB27B
220236_at	6.27E-03	1.70	up	Hs.655245	PDPR

226225_at	6.28E-03	1.73	down	Hs.593171	MCC
244324_at	6.33E-03	2.08	down	Hs.208701	C18orf54
242805_at	6.92E-03	2.28	up		
208304_at	6.94E-03	2.66	down	Hs.506190	CCR3
203747_at	6.97E-03	1.80	down	Hs.234642	AQP3
1554612_at	7.08E-03	1.73	up	Hs.478868	KIAA0226
228535_at	7.20E-03	1.60	down	Hs.531879	RAD1
239105_at	7.76E-03	1.52	up	Hs.121525	
213942_at	7.88E-03	2.60	down	Hs.593645	MEGF6
235157_at	8.02E-03	1.88	up	Hs.664716	
219737_s_at	8.08E-03	1.95	down	Hs.654709	PCDH9
32128_at	8.13E-03	2.98	up	Hs.143961	CCL18
1570253_a_at	8.25E-03	1.67	up	Hs.159013	RHEBL1
231926_at	8.34E-03	1.51	down	Hs.654639	EPS15L1
1554007_at	8.43E-03	1.65	down	Hs.166348	
243764_at	8.56E-03	1.60	down	Hs.177164	VSIG1
220532_s_at	8.60E-03	2.63	up	Hs.647090	TMEM176B
213739_at	8.67E-03	1.68	down	Hs.458447	
1560827_at	8.70E-03	2.37	down	Hs.572321	
235276_at	8.71E-03	1.56	up	Hs.546467	EPSTI1
205407_at	8.97E-03	1.58	down	Hs.388918	RECK
230168_at	9.05E-03	1.62	down	Hs.509213	
229684_s_at	9.09E-03	1.63	up	Hs.173001	ZNF644
227803_at	9.20E-03	2.33	down	Hs.35198	ENPP5
242343_x_at	9.24E-03	1.73	up		
1553660_at	9.35E-03	1.73	up	Hs.669039	HUS1B
235363_at	9.45E-03	1.63	down	Hs.28360	
209504_s_at	9.58E-03	1.60	down	Hs.445489	PLEKHB1
202454_s_at	9.59E-03	1.79	down	Hs.118681	ERBB3
202953_at	9.73E-03	3.54	up	Hs.8986	C1QB
231171_at	9.77E-03	1.68	down	Hs.652420	
211187_at	1.00E-02	2.54	up		
209924_at	1.02E-02	3.03	up	Hs.143961	CCL18
238554_at	1.02E-02	1.60	down	Hs.461131	CYB5B
207509_s_at	1.02E-02	1.99	down	Hs.43803	LAIR2
228124_at	1.02E-02	1.54	up	Hs.441550	ABHD12
200962_at	1.02E-02	1.89	down	Hs.469473	RPL31
1554533_at	1.05E-02	3.22	up	Hs.408903	C2
203411_s_at	1.05E-02	1.60	up	Hs.594444	LMNA
243794_at	1.09E-02	2.50	up	Hs.119922	
218631_at	1.11E-02	1.56	up	Hs.23918	AVPI1
212731_at	1.12E-02	1.80	down	Hs.530199	ANKRD46
217367_s_at	1.14E-02	1.51	down	Hs.380133	ZHX3
228488_at	1.14E-02	1.69	down	Hs.369819	TBC1D16
202411_at	1.15E-02	4.25	up	Hs.532634	IFI27
223862_at	1.16E-02	1.58	down	Hs.590080	GHRL

212859_x_at	1.17E-02	1.66	up	Hs.534330	MT1E
231152_at	1.22E-02	1.58	up	Hs.445036	FLJ20309
209039_x_at	1.23E-02	1.57	down	Hs.523774	EHD1
1566043_at	1.27E-02	2.08	up	Hs.684729	YR76A02
1559648_at	1.28E-02	2.28	down	Hs.152595	LOC100128420
228513_at	1.29E-02	1.69	down	Hs.460574	LOC124446
215295_at	1.33E-02	2.04	down	Hs.307720	DTNB
215623_x_at	1.39E-02	1.55	up	Hs.58992	SMC4
231458_at	1.40E-02	2.51	down	Hs.599340	
239343_at	1.40E-02	1.85	down	Hs.129828	LOC728705
231817_at	1.41E-02	1.57	down	Hs.431081	USP53
1558859_at	1.44E-02	1.65	down		LOC222159
215617_at	1.44E-02	1.52	up	Hs.120323	LOC26010
242672_at	1.44E-02	1.86	up	Hs.665622	
233511_at	1.46E-02	1.52	up	Hs.127379	
243581_at	1.47E-02	2.00	up	Hs.659284	LOC646470
216230_x_at	1.48E-02	1.59	down	Hs.498173	SMPD1
243318_at	1.49E-02	1.50	up	Hs.632447	WDR42A
238030_at	1.50E-02	1.52	down		
241851_x_at	1.55E-02	2.13	up	Hs.633029	LOC100130429
203915_at	1.56E-02	3.05	up	Hs.77367	CXCL9
204415_at	1.58E-02	1.84	up	Hs.511731	IFI6
205683_x_at	1.58E-02	3.27	down	Hs.405479	TPSAB1 /// TPSB2
238909_at	1.60E-02	1.92	up	Hs.143873	S100A10
222962_s_at	1.62E-02	2.06	down	Hs.198363	MCM10
235594_at	1.62E-02	1.52	up	Hs.591680	SCYE1
232102_at	1.64E-02	2.63	down	Hs.149487	METTL6
244784_at	1.65E-02	2.14	up	Hs.468226	DHX57
219635_at	1.67E-02	1.53	down	Hs.654967	ZNF606
216147_at	1.67E-02	2.61	up	Hs.675702	
210095_s_at	1.68E-02	1.81	down	Hs.450230	IGFBP3
46665_at	1.68E-02	1.53	down	Hs.516220	SEMA4C
202342_s_at	1.69E-02	1.83	down	Hs.435711	TRIM2
230657_at	1.69E-02	1.62	up	Hs.668532	
215238_s_at	1.70E-02	2.02	up	Hs.654825	DOCK9
212086_x_at	1.72E-02	1.94	up	Hs.594444	LMNA
228213_at	1.72E-02	1.55	down	Hs.524280	H2AFJ
225688_s_at	1.73E-02	1.59	down	Hs.477114	PHLDB2
226258_at	1.74E-02	1.51	down	Hs.591146	AMN1
204083_s_at	1.76E-02	1.79	down	Hs.300772	TPM2
1558700_s_at	1.78E-02	1.64	down	Hs.18103	ZNF260
224548_at	1.79E-02	1.53	down	Hs.434828	HES7
232503_at	1.79E-02	2.36	up		
223815_at	1.82E-02	1.55	up	Hs.569713	CCDC45
225270_at	1.83E-02	1.80	down	Hs.388613	NEO1
1569948_at	1.84E-02	1.85	up	Hs.685028	

225353_s_at	1.87E-02	3.95	up	Hs.467753	C1QC
216474_x_at	1.89E-02	2.95	down	Hs.405479	TPSAB1 /// TPSB2
1562697_at	1.90E-02	1.56	down	Hs.434746	LOC339988
211719_x_at	1.90E-02	2.59	up	Hs.203717	FN1
218950_at	1.91E-02	1.68	down	Hs.25277	CENTD3
209815_at	1.91E-02	1.51	down	Hs.494538	PTCH1
1556123_a_at	1.92E-02	1.53	down	Hs.637720	
227195_at	1.93E-02	1.83	up	Hs.195710	ZNF503
214945_at	1.95E-02	1.85	down	Hs.646916	FAM153
209737_at	1.97E-02	1.70	down	Hs.654788	MAGI2
216442_x_at	1.97E-02	2.75	up	Hs.203717	FN1
1562386_s_at	1.98E-02	2.04	down	Hs.401045	ZNF501
76897_s_at	1.98E-02	1.79	up	Hs.522351	FKBP15
243005_at	2.02E-02	1.56	up	Hs.458593	
210189_at	2.02E-02	1.52	down	Hs.690634	HSPA1L
207175_at	2.02E-02	2.47	down	Hs.80485	ADIPOQ
1561017_at	2.04E-02	1.82	down	Hs.684677	
223044_at	2.06E-02	1.59	down	Hs.643005	SLC40A1
215443_at	2.08E-02	2.53	down	Hs.160411	TSHR
225248_at	2.09E-02	1.62	down	Hs.700687	SPPL2B
231889_at	2.09E-02	1.61	up	Hs.148078	UBR4
217657_at	2.11E-02	2.42	down	Hs.303787	BCAP29
204430_s_at	2.13E-02	4.47	up	Hs.530003	SLC2A5
238535_at	2.17E-02	1.92	down	Hs.597323	CEP152
238731_at	2.20E-02	2.27	up	Hs.631789	SETDB2
244331_at	2.20E-02	2.36	up	Hs.609761	
208302_at	2.23E-02	2.53	down	Hs.158320	HMHB1
223609_at	2.25E-02	2.33	down	Hs.381089	ROPN1L
242695_at	2.28E-02	1.58	up	Hs.634325	
208112_x_at	2.28E-02	1.51	down	Hs.523774	EHD1
204745_x_at	2.30E-02	1.67	up	Hs.433391	MT1G
220666_at	2.32E-02	1.93	up		
228713_s_at	2.33E-02	1.65	up	Hs.18788	HSD17B14
1553810_a_at	2.35E-02	1.68	down	Hs.591308	KIAA1524
1556060_a_at	2.37E-02	1.71	up	Hs.656721	KIAA1702
208581_x_at	2.43E-02	1.52	up	Hs.374950	
202767_at	2.43E-02	1.63	up	Hs.532492	
234761_at	2.44E-02	1.53	down	Hs.679413	
241582_at	2.45E-02	1.65	down	Hs.587446	
241438_at	2.45E-02	2.34	up	Hs.683735	
241400_at	2.45E-02	1.71	up		
235874_at	2.45E-02	2.17	down	Hs.98381	PRSS35
235875_at	2.46E-02	2.92	up	Hs.323878	
209210_s_at	2.47E-02	2.69	up	Hs.712571	FERMT2
1554500_a_at	2.49E-02	2.24	down	Hs.655739	RGS7
239655_at	2.49E-02	1.87	up	Hs.661731	

211514_at	2.49E-02	2.18	up	Hs.6874	RIPK5
240207_at	2.51E-02	1.57	up	Hs.664722	
209485_s_at	2.53E-02	2.43	up	Hs.370725	OSBPL1A
1568720_at	2.56E-02	1.67	down	Hs.351906	ZNF506
226187_at	2.57E-02	1.73	up	Hs.656883	FLJ23044
1559042_at	2.60E-02	2.04	down	Hs.493668	NDUFB6
238962_at	2.61E-02	1.67	down	Hs.399952	ZNF681
236805_at	2.64E-02	1.89	down	Hs.632077	C9orf96
222916_s_at	2.65E-02	1.58	down	Hs.471851	HDLBP
1556583_a_at	2.65E-02	2.41	up	Hs.31961	SLC8A1
219976_at	2.68E-02	1.80	down	Hs.378836	HOOK1
227462_at	2.68E-02	2.07	up	Hs.482910	
210495_x_at	2.68E-02	2.49	up	Hs.203717	FN1
234685_x_at	2.69E-02	2.17	down	Hs.307018	KRTAP4-9
202075_s_at	2.70E-02	2.71	up	Hs.439312	PLTP
243067_at	2.71E-02	1.93	up	Hs.613623	
237112_at	2.74E-02	2.03	up		
1554609_at	2.74E-02	1.51	down	Hs.82502	POLD3
204727_at	2.75E-02	1.67	down	Hs.385998	WDHD1
210354_at	2.76E-02	1.74	up	Hs.856	IFNG
241788_x_at	2.79E-02	2.28	up	Hs.663702	
232180_at	2.79E-02	1.63	down	Hs.516217	UGP2
203355_s_at	2.80E-02	1.59	up	Hs.434255	PSD3
243286_at	2.84E-02	1.55	up	Hs.657069	
212464_s_at	2.84E-02	2.44	up	Hs.203717	FN1
227939_s_at	2.85E-02	2.24	up	Hs.699226	
233193_x_at	2.86E-02	1.72	up	Hs.533723	
235706_at	2.88E-02	1.59	up	Hs.654387	CPM
227578_at	2.90E-02	2.18	down	Hs.594042	LOC100128191
201785_at	2.90E-02	3.64	up	Hs.78224	RNASE1
214701_s_at	2.90E-02	2.08	up	Hs.203717	FN1
211018_at	2.90E-02	1.82	up	Hs.708002	LSS
235868_at	2.90E-02	1.54	up	Hs.500842	MGEA5
236313_at	2.93E-02	2.06	down	Hs.72901	CDKN2B
228806_at	2.95E-02	3.37	down	Hs.256022	RORC
1553849_at	2.95E-02	1.65	down	Hs.679457	CCDC26
226435_at	2.96E-02	1.56	down	Hs.655583	PAPLN
237865_x_at	2.96E-02	1.76	up		
1564121_at	2.98E-02	1.77	up	Hs.677395	FLJ23135
243209_at	3.03E-02	1.76	down	Hs.473058	KCNQ4
244633_at	3.04E-02	1.64	up	Hs.662815	
1552386_at	3.04E-02	2.29	down	Hs.547697	C5orf29
1562698_x_at	3.05E-02	1.53	down	Hs.434746	
235104_at	3.07E-02	2.12	up	Hs.591249	ERAP2
239546_at	3.08E-02	2.04	down	Hs.697104	LOC100131053
1564424_at	3.08E-02	1.74	up	Hs.685329	

218232_at	3.10E-02	3.08	up	Hs.632379	C1QA
207440_at	3.12E-02	1.58	up	Hs.21899	SLC35A2
212096_s_at	3.13E-02	2.06	down	Hs.7946	MTUS1
207008_at	3.14E-02	2.30	down	Hs.846	IL8RB
215985_at	3.14E-02	1.57	down	Hs.653168	C6orf12
223680_at	3.15E-02	1.70	down	Hs.334518	ZNF607
220220_at	3.15E-02	1.93	up	Hs.675446	LRRC37A4
240211_at	3.15E-02	1.72	down	Hs.662509	LOC100130468
239650_at	3.16E-02	2.30	down	Hs.537329	NAP5
213709_at	3.16E-02	1.81	down	Hs.4276	BHLHB9
234520_at	3.16E-02	2.06	down		
1561757_a_at	3.17E-02	2.23	down	Hs.665667	LOC283352
232664_at	3.17E-02	1.67	down	Hs.289062	FLJ12334
205819_at	3.18E-02	3.10	up	Hs.67726	MARCO
209230_s_at	3.18E-02	2.76	up	Hs.513463	NUPR1
215854_at	3.21E-02	2.38	up	Hs.658795	
221841_s_at	3.22E-02	1.52	up	Hs.376206	KLF4
241826_x_at	3.22E-02	1.75	down	Hs.124384	
239232_at	3.23E-02	1.81	up	Hs.658922	MSI2
210694_s_at	3.23E-02	2.06	down	Hs.27695	MID1
243683_at	3.24E-02	1.77	up	Hs.326387	MORF4L2
239502_at	3.27E-02	1.82	down	Hs.560456	
233867_at	3.27E-02	1.91	up	Hs.610960	
228718_at	3.28E-02	1.62	down	Hs.296731	ZNF44
234282_at	3.29E-02	1.68	down	Hs.543773	
1553992_s_at	3.32E-02	1.59	down		NBR2
244619_at	3.33E-02	1.52	up	Hs.653214	LOC646626
232679_at	3.35E-02	1.57	down	Hs.655296	
220716_at	3.35E-02	2.41	down	FLJ12595	
228698_at	3.37E-02	2.13	down	Hs.709543	SOX7
222577_at	3.38E-02	1.90	down	Hs.368866	CCDC90B
243496_at	3.41E-02	1.63	up	Hs.406799	RAB18
235424_at	3.44E-02	1.69	down	Hs.655632	
229970_at	3.45E-02	1.55	down	Hs.63841	KBTBD7
214820_at	3.46E-02	1.50	down	Hs.654740	BRWD1
239951_at	3.47E-02	2.15	down	Hs.432535	
238687_x_at	3.47E-02	2.56	up	Hs.5327	ZNF770
220266_s_at	3.48E-02	1.83	up	Hs.376206	KLF4
221212_x_at	3.48E-02	1.60	up	Hs.189920	PBRM1
1553562_at	3.52E-02	1.64	up	Hs.405667	CD8B
206622_at	3.56E-02	2.18	down	Hs.182231	TRH
223958_s_at	3.56E-02	1.75	up	Hs.525445	DNAL1
232416_at	3.59E-02	2.60	up	Hs.655747	BRUNOL5
244841_at	3.59E-02	1.86	down	Hs.595540	SEC24A
232738_at	3.61E-02	2.11	down		
209263_x_at	3.62E-02	1.55	up	Hs.654836	TSPAN4

243108_at	3.63E-02	1.66	up		
204612_at	3.65E-02	1.72	down	Hs.433700	PKIA
217023_x_at	3.66E-02	2.92	down	Hs.405479	TPSAB1 /// TPSB2
214716_at	3.66E-02	1.60	up	Hs.146551	BMP2K
205390_s_at	3.67E-02	2.00	down	Hs.654438	ANK1
219759_at	3.68E-02	2.24	up	Hs.591249	ERAP2
204204_at	3.70E-02	1.74	up	Hs.24030	SLC31A2
214702_at	3.71E-02	2.25	up	Hs.203717	FN1
213692_s_at	3.72E-02	1.62	up	Hs.524368	VDR
203819_s_at	3.73E-02	2.54	up	Hs.700696	IGF2BP3
1559535_s_at	3.75E-02	1.53	down	Hs.314414	
201159_s_at	3.76E-02	1.72	down	Hs.532790	NMT1
217663_at	3.76E-02	1.78	down	Hs.235992	
217184_s_at	3.76E-02	1.85	down	Hs.434481	LTK
217566_s_at	3.79E-02	1.82	up	Hs.438265	TGM4
224276_at	3.81E-02	1.81	down	Hs.435774	ZNF33A
224526_at	3.82E-02	1.99	down	Hs.711457	LOC100132319
1564691_at	3.83E-02	2.44	down	Hs.525914	LOC100128594
228407_at	3.85E-02	1.50	up	Hs.12923	SCUBE3
1557993_at	3.85E-02	1.85	up	Hs.586906	
210652_s_at	3.86E-02	1.70	down	Hs.112949	C1orf34
239302_s_at	3.87E-02	1.63	down	Hs.587092	
213317_at	3.87E-02	1.74	down	Hs.485489	CLIC5
241888_at	3.90E-02	1.70	down	Hs.207982	
244026_at	3.93E-02	2.06	up	Hs.604381	
227083_at	3.95E-02	1.84	down	Hs.13205	B3GALTL
205803_s_at	3.95E-02	1.60	down	Hs.250687	TRPC1
219617_at	3.96E-02	1.52	down	Hs.468349	C2orf34
31874_at	3.96E-02	1.64	up	Hs.322852	GAS2L1
234056_at	3.97E-02	1.76	down	Hs.636089	
243541_at	3.99E-02	2.76	up	Hs.55378	IL31RA
205738_s_at	4.00E-02	2.27	up	Hs.657242	FABP3
1554476_x_at	4.02E-02	1.73	down	Hs.235167	ZNF808
1559258_a_at	4.04E-02	1.89	up	Hs.97892	CXorf61
1554108_at	4.07E-02	1.57	down		
234212_at	4.09E-02	2.28	up	Hs.699451	ACTR2
216298_at	4.11E-02	1.69	down		LOC648852
229065_at	4.12E-02	1.79	down	Hs.158748	SLC35F3
177_at	4.13E-02	1.82	down	Hs.382865	PLD1
232397_at	4.15E-02	1.64	down	Hs.191841	
236545_at	4.17E-02	1.58	up	Hs.662933	
206974_at	4.21E-02	1.51	down	Hs.34526	CXCR6
209871_s_at	4.21E-02	1.83	down	Hs.618112	APBA2
1554980_a_at	4.21E-02	1.82	up	Hs.460	ATF3
208711_s_at	4.23E-02	1.64	up	Hs.523852	CCND1
205181_at	4.25E-02	1.50	down	Hs.712570	ZNF193

204955_at	4.28E-02	1.97	down	Hs.15154	SRPX
1562058_at	4.30E-02	1.83	down	Hs.129111	FLJ37414
1552789_at	4.30E-02	1.51	down	Hs.592561	SEC62
236655_at	4.32E-02	1.78	down	Hs.368433	TPD52
1554672_at	4.33E-02	1.98	up	Hs.659165	TTC26
216248_s_at	4.34E-02	2.34	up	Hs.563344	NR4A2
1562888_at	4.36E-02	1.58	up		GLB1L3
204452_s_at	4.37E-02	1.56	up	Hs.94234	FZD1
243361_at	4.37E-02	2.93	up	Hs.519347	SFRS12
241843_at	4.38E-02	1.62	up	Hs.658237	SNORA28
200986_at	4.40E-02	2.22	up	Hs.384598	SERPING1
1554391_at	4.40E-02	1.92	up	Hs.607889	TBK1
243771_at	4.46E-02	1.51	up		
1553994_at	4.52E-02	2.02	down	Hs.153952	NT5E
203440_at	4.53E-02	2.45	down	Hs.464829	CDH2
217628_at	4.53E-02	1.74	down	Hs.485489	CLIC5
228295_at	4.55E-02	1.62	up	Hs.280951	WDR59
242413_at	4.56E-02	1.58	up		
238796_at	4.56E-02	1.69	up	Hs.661606	
1559436_x_at	4.57E-02	1.51	up	Hs.684047	
238069_at	4.60E-02	1.52	down	Hs.535847	
212831_at	4.63E-02	2.45	up	Hs.494977	MEGF9
220080_at	4.64E-02	1.51	down	Hs.710714	FBXL8
1569952_x_at	4.67E-02	1.71	up	Hs.454036	
213994_s_at	4.70E-02	1.99	down	Hs.705394	SPON1
227839_at	4.71E-02	1.94	down	Hs.458312	MBD5
237640_at	4.71E-02	1.70	up	Hs.558541	C14orf138
201430_s_at	4.72E-02	1.99	up	Hs.519659	DPYSL3
223666_at	4.73E-02	1.51	up	Hs.316890	SNX5
1569385_s_at	4.73E-02	1.63	up	Hs.367639	TET2
211469_s_at	4.75E-02	1.57	down	Hs.34526	CXCR6
238427_at	4.76E-02	1.76	down	Hs.511816	GRPEL2
244728_at	4.79E-02	1.51	down	Hs.634550	
242094_at	4.79E-02	2.33	down	Hs.669515	YR40C10
226697_at	4.80E-02	1.63	up	Hs.476517	FAM114A1
1569139_s_at	4.81E-02	1.73	down	Hs.143314	FAM53A
202672_s_at	4.85E-02	1.91	up	Hs.460	ATF3
1564709_at	4.86E-02	1.67	up	Hs.434185	LOC286238
234907_x_at	4.87E-02	1.53	up	Hs.661106	
230192_at	4.93E-02	1.59	down	Hs.436922	TRIM13
241159_x_at	4.94E-02	1.73	up	Hs.665631	
211180_x_at	4.95E-02	1.67	up	Hs.149261	RUNX1
1564360_a_at	4.96E-02	2.08	up	Hs.661076	LOC339260
241687_at	4.97E-02	1.54	down	Hs.155775	
236437_at	4.99E-02	1.66	up	Hs.666117	

[1] FC, Fold Change is the ratio of mean expression levels for each group.
[2] Regulation is the whether the mean expression level of the extended-to-be group is “up” or “down” compared to the persistent group.
